# Asymptomatic intramural esophageal dissection: A case report and literature review

**DOI:** 10.1097/MD.0000000000042632

**Published:** 2025-05-30

**Authors:** Gong Cheng, Li Wang, Jiateng You, Liangliang Cheng, Fan Yang, Xiaolu Wang, Fang He, Changjin Li, Xiaoguo Wei

**Affiliations:** a Department of Gastroenterology, Gansu Provincial Hospital, Lanzhou, China; b Department of Hematology, Gansu Provincial Hospital of TCM, Lanzhou, China; c The Second Clinical Medical College, Lanzhou University, Lanzhou, China; d Vanke School of Public Health, Tsinghua University, Beijing, China; e Department of Radiation Oncology, Medical Faculty Mannheim, Heidelberg University, Mannheim, Germany.

**Keywords:** asymptomatic, case, conservative treatment, intramural esophageal dissection

## Abstract

**Rationale:**

Intramural esophageal dissection (IED) is a rare condition, with asymptomatic cases not previously reported. This report aims to highlight an incidental finding of IED in a patient undergoing routine gastroscopy.

**Patient concerns:**

A 41-year-old male with decompensated liver cirrhosis underwent gastroscopy, revealing IED without prior symptoms or history of esophageal surgery or injury.

**Diagnoses:**

IED.

**Interventions:**

Conservative treatment was chosen due to the patient’s poor coagulation function and low platelet count, avoiding potential complications such as swallowing difficulties or hematemesis.

**Outcomes:**

The patient remained asymptomatic and stable during follow-up.

**Lessons:**

This case underscores the importance of considering IED in differential diagnoses, even in asymptomatic patients, and highlights conservative management’s efficacy in specific scenarios.

## 1. Introduction

Intramural esophageal dissection (IED) is a rare condition characterized by the separation of the esophageal mucosa from the underlying muscle. While symptomatic cases have been documented, asymptomatic IED remains unreported. This case highlights an incidental discovery of IED during a routine examination in a patient with decompensated liver cirrhosis.

## 2. Case presentation

### 2.1. Patient information

A 41-year-old male with weakness and extremity edema. The patient had a history of hepatitis B for 18 years and was diagnosed during a hospitalization for a car accident.

### 2.2. Clinical findings

The patient presented with weakness, edema of the extremities, and jaundice. Physical examination showed stable vital signs and mild jaundice.

### 2.3. Timeline

Symptoms developed a month prior to presentation.

### 2.4. Diagnostic assessment

**Figure 1.**
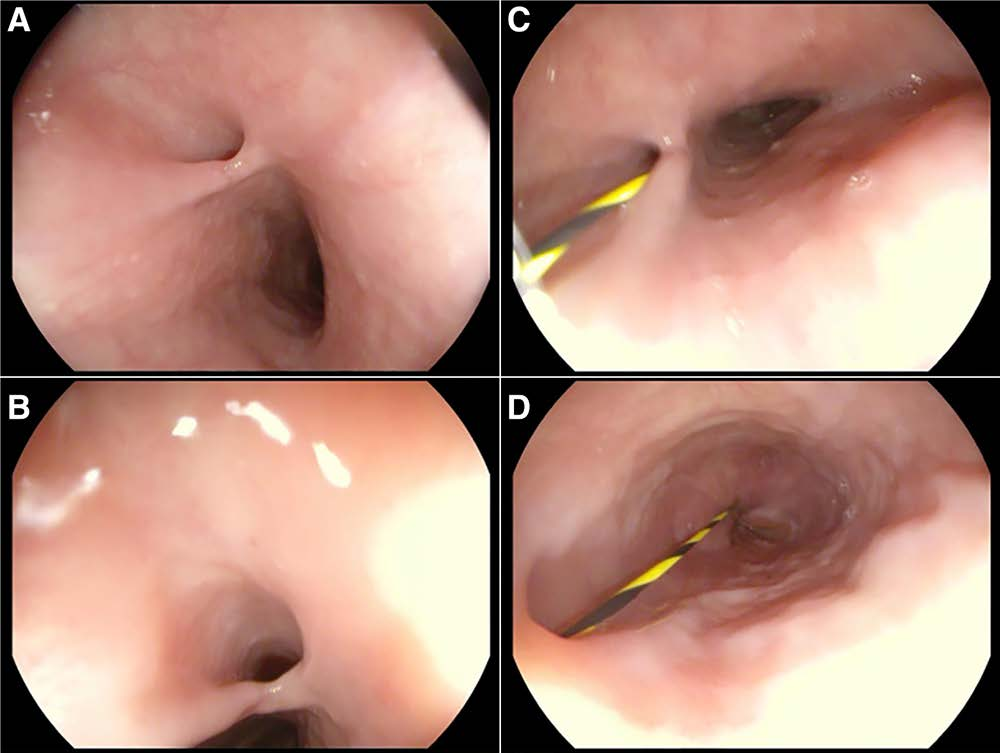
Gastroscopy showed the opening of the false lumen at 15 cm from the incisor and the outlet at 40 cm from the incisor.

**Figure 2.**
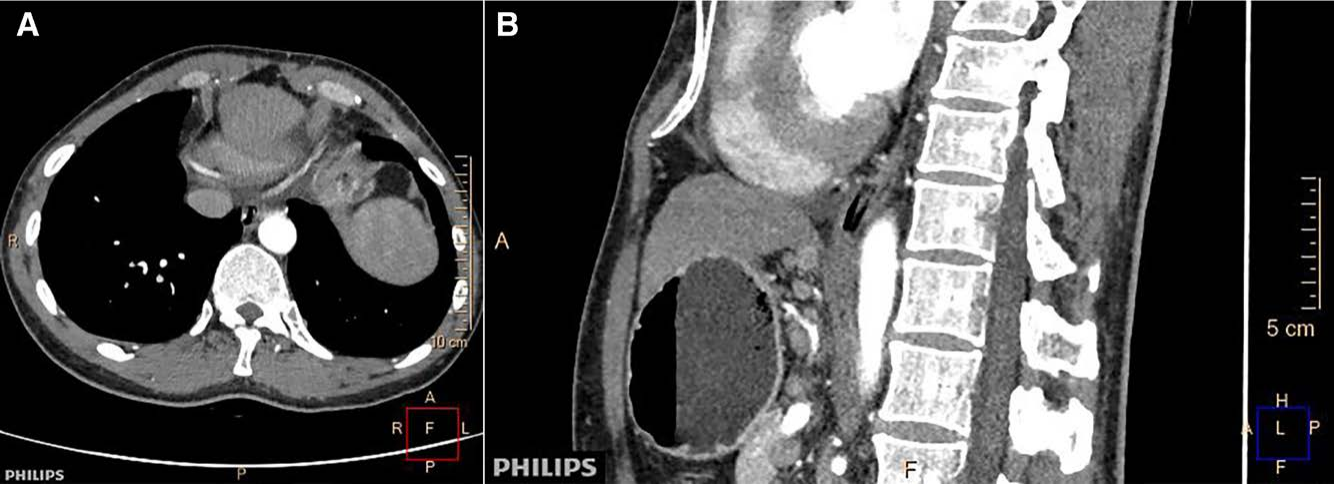
Enhanced computed tomography of the chest confirmed the dissection with air within the esophageal wall.

The laboratory finding shows poor coagulation, prothrombin time activity of 56%, prothrombin time of 18.2 seconds, and activated partial thromboplastin time of 48 seconds. A platelet count of 70,000 per mm³ and blood ammonia of 167.51 μmol/L. An hepatitis B virus deoxyribonucleic acid of 5.67 × 10^5^ IU/ml. We performed an gastroscopy to evaluate whether the patient has esophageal and gastric varices. Gastroscopy did not find esophageal and gastric varices but revealed a fistula-like structure 15 cm from the incisors. Upon further entry, another fistula-like structure was found at 40 cm from the incisors. We tried to verify that the 2 openings of the fistula-like structure communicated by injecting methylene blue. But it failed because of operational difficulties. Therefore, we used a zebra guide wire to enter from 15 cm and found that the guide wire was exported from the 40 cm opening (Fig. 1). Enhanced computed tomography (CT) of the chest confirmed the dissection with air within the esophageal wall, but no transmural perforation (Fig. 2).

### 2.5. Therapeutic intervention

Due to poor coagulation function and low platelet count in patients with decompensated cirrhosis after hepatitis B, the treatment was mainly conservative treatment such as antiviral treatment. Because the patient had no symptoms such as chest pain, dysphagia, and hematemesis, the IED was observed conservatively.

### 2.6. Follow-up and outcomes

The patient remained asymptomatic and stable during follow-up.

## 3. Discussion

IED is a rare disease.^[1]^ It was 1st reported by Marks IN and Keet AD in 1968.^[2]^ The etiology of IED is still unclear. According to the current reports, the possible causes of IED may include the following aspects. First, IED may occur due to severe vomiting. A sudden rise in esophageal pressure during vomiting causes a tear in the esophageal mucosa. If the tear is limited, it will lead to Mallory–Weiss syndrome (MWS)^[3]^ or IED,^[4]^ and in severe cases, spontaneous esophageal rupture (Boerhaave syndrome).^[5]^ Of course, medical procedures such as endoscopy,^[6]^ gastric tube insertion,^[7]^ and other procedures may also cause the above injuries. Secondly, some patients have coagulation dysfunction, including liver disease, taking anticoagulant or antiplatelet drugs, blood system diseases, such as platelet abnormalities (reduction in quantity and dysfunction), and bleeding tendency may lead to or promote the occurrence of esophageal intramural dissection.^[8]^ Related studies suggest^[9]^ that IED may be one of the complications of eosinophilic esophagitis (EOE), and most of these patients have spontaneous IED without a history of related esophageal mucosal injury. In summary, there are 2 hypotheses for the pathogenesis of IED: Spontaneous esophageal intramural hematoma leading to dissection and The mucosal tear leads to dissection.^[10]^

We searched Pubmed for case reports up to September 10, 2024, and a total of 49 articles^[1,4,6,7,9–53]^ with a total of 54 cases with a definite diagnosis of IED were retrieved (Table S1, Supplemental Digital Content, http://links.lww.com/MD/P55). The cases included 32 (59%) men and 22 (41%) women, with ages ranging from 2 months to 87 years. Among these cases, 3 cases were located in the cervical esophagus, 17 cases in the thoracic esophagus, 2 cases in the abdominal esophagus, 7 cases in the cervical and thoracic esophagus, and 12 cases in the thoracic and abdominal esophagus. The dissection occupied the whole esophagus in 8 cases, and one of them involved the fundus of the stomach. Among these cases, IED was 1st detected by CT in 26 cases, by gastroscopy in 19 cases, by upper gastrointestinal radiography in 7 cases, and by transabdominal color Doppler ultrasound in 1 case.

**Figure 3.**
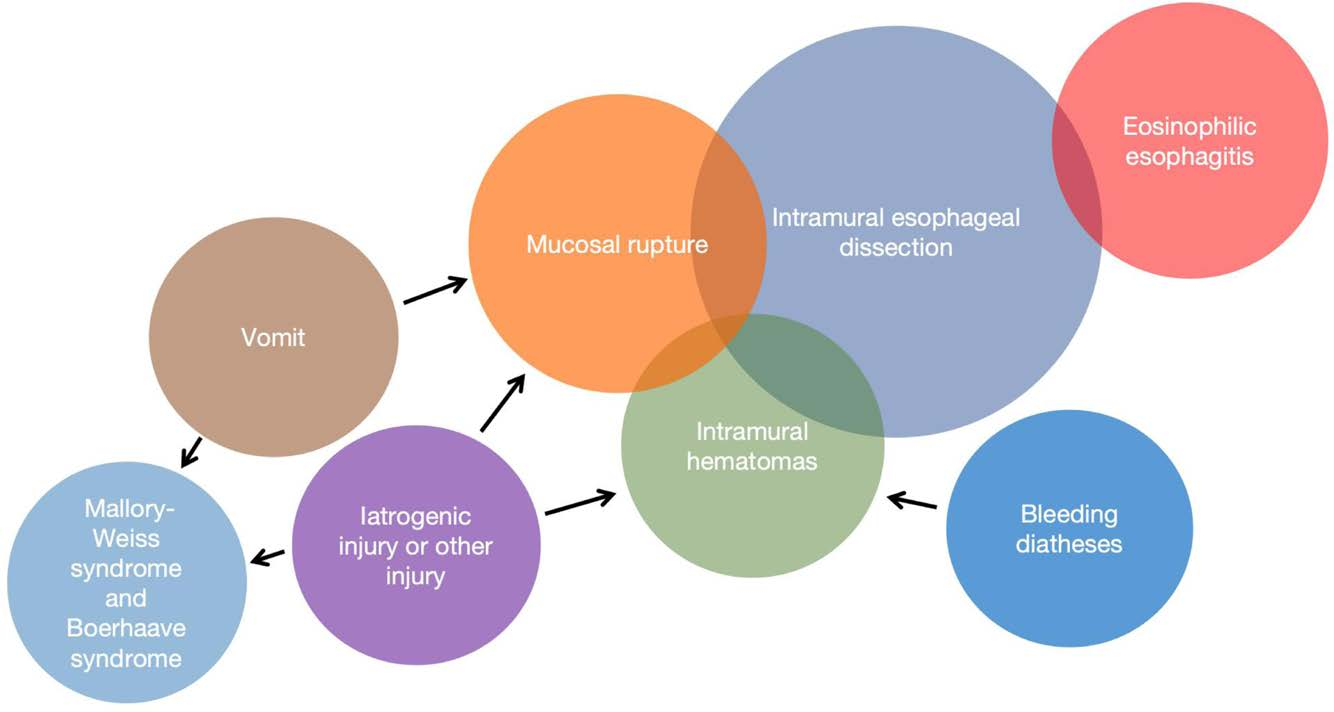
Etiologic diagram of IED. IED = intramural esophageal dissection.

The etiology of IED is not clear. According to its pathogenesis, diseases that can lead to tears of the esophageal mucosa and/or spontaneous hematoma may lead to IED. Our statistics show that mechanical injury is an important cause of IED, which includes iatrogenic manipulation and vomiting. Ten percent of IED occur in the presence of severe vomiting.^[4,30,38,39,45]^ These patients may have a history of alcohol consumption or suffer from injury or tear of the esophageal mucosa caused by vomiting secondary to intestinal obstruction. Iatrogenic injury is the most common cause of esophageal intramural dissection. These invasive procedures include the endoscopic examination and treatment,^[6,29,33,34]^ peroral endoscopic myotomy,^[49]^ transesophageal echocardiography,^[26]^ nasogastric tube or nasobiliary tube insertion,^[7,13,15]^ and surgical stapler injury.^[25]^ Most of these iatrogenic procedures were esophageal intraluminal procedures. Abnormal coagulation function, including liver disease,^[1]^ thrombocytopenia, and the use of anticoagulant and antiplatelet drugs,^[10,21,22,38,47]^ can lead to spontaneous hematoma formation of the esophageal mucosa and cause or promote the occurrence of intramural dissection of the esophagus. Pajot et al^[52]^ reported a case of IED possibly after chest compression. This patient was complicated with coronary heart disease, received dual antiplatelet therapy, and chest compression was performed due to cardiac arrest. This may also indicate that IED is a disease caused by 1 or more factors under specific conditions. Pharyngeal diseases may also lead to IED. According to literature review, IED was found in 1 case of pharyngeal abscess^[21]^ and 1 case of pharyngeal squamous cell carcinoma treated with radiotherapy and chemotherapy.^[17]^ Eosinophilic esophagitis is a very important cause of IED, and 15% of IED patients have EoE. EoE is a long-term mucosal inflammation that reduces the integrity of the epithelial barrier, and esophageal fibrosis leading to esophageal stenosis increases the risk of physical damage to the esophagus.^[54]^ Diet may also be one of the causes of IED. In some rare cases, IED patients consume whole grains or alcohol before the onset of the disease, and these patients have no vomiting or definite history of esophageal injury^[46,48]^ (Fig. 3).

In the past, the definition of IED was not clear, and it was once confused with intramural esophageal hematoma. However, it is believed that intramural hematoma is not equivalent to intramural dissection of the esophagus, which depends on whether the submucosal false lumen of the esophagus is present or not, and whether the false lumen is communicating with the true lumen of the esophagus. In a rare case, the patient took betel nut juice which caused an intramural hematoma of the esophagus. After conservative treatment, the hematoma disappeared without the formation of IED.^[55]^ Thus, there are 2 possible developmental outcomes of intramural hematoma formation: hematoma absorption or the development of intramural dissection. The clinical manifestations such as chest pain, dysphagia, and hematemesis do not have typical characteristics, and symptoms cannot be used as a necessary condition for the diagnosis of IED. There are various types of esophageal mucosal injury. IED, MWS, and Boerhaave syndrome are different types of esophageal mucosal injury. The injury of IED is characterized by the presence of true and false lumen, esophageal mucosal injury, submucosal dissection, and septal layer between true and false lumen, followed by full-thickness perforation of the esophageal wall and mediastinal abscess. However, MWS is a non-penetrating mucosal laceration of the distal esophagus near the gastroesophageal junction without false lumen, which is a non-penetrating mucosal laceration with bleeding as the main clinical manifestation.^[56]^ In contrast, Boerhaave syndrome is a transmural esophageal perforation, which may be iatrogenic, traumatic, caused by a foreign body, associated with other pathologies, or spontaneous. Iatrogenic perforation was the most common (accounting for 52.1% of cases).^[56]^ Unlike MWS tears, the latter are non-penetrating mucosal lacerations of the distal esophagus near the gastroesophageal junction.^[56]^ Both are more common in men with excessive alcohol consumption and are associated with esophageal barotrauma. However, hematemesis is common in MWS but not in Boerhaave syndrome.^[57]^ We suggest that the 3 different patterns of esophageal injury, MWS, Boerhaave syndrome and IED, may transpose into each other. Case reports have confirmed the transition of MWS to Boerhaave syndrome.^[57]^ Wang X et al^[31]^ reported a case of esophageal perforation secondary to endoscopic resection of esophageal leiomyoma. The patient was confirmed to have esophageal perforation by postoperative CT scan, and upper gastrointestinal radiography after 7 days of treatment suggested the presence of IED. According to our retrospective statistics, some patients with IED had secondary esophageal perforation, mediastinal emphysema, mediastinitis, hemothorax, and other complications.^[18,20,22]^

IED has similar clinical manifestations to MWS, Boerhaave syndrome, and intramural esophageal hematoma. The most common clinical manifestations of IED were retrosternal pain, odynophagia, and hematemesis, which were consistent with our statistics. In a review of previous studies, 29 (54%) patients had chest pain, 33 (61%) had odynophagia and/or dysphagia, and 19 (35%) had hematemesis. Therefore, we proposed the IED triad of “chest pain, odynophagia/dysphagia, and hematemesis.” Of course, the absence of typical symptoms in this patient suggests that covert IED also exists, and this is the 1st report of covert IED. In addition to esophageal lesions, IED patients may also have complications, such as mediastinal emphysema, esophageal abscess, empyema, esophageal pleural fistula and other early complications. Esophageal stenosis is a common and serious late complication of IED.

CT scan, gastroscopy and upper gastrointestinal radiography are very important for the diagnosis of IED. Most IED patients were diagnosed by these 3 methods. Neck and chest CT of IED can show double-barrel esophagus,^[58]^ namely true lumen and false lumen. It may also only indicate the presence of free air in the esophageal wall. Upper gastrointestinal radiography showed double-tube dilatation of the esophageal lumen caused by intramuscular dissection.^[2]^ “Double barrel esophagus sign”^[58]^ is a radiological feature of IED without perforation. The landmark represents the 2 lumens of the esophagus, the true mucosal lumen and the pseudomucosal lumen, which are separated by a mucosal band. Gastroscopy is the gold standard for the diagnosis of IED. Gastroscopy shows the true lumen and false lumen of the esophagus, which are separated by mucosal bridge and mucosal flap. Therefore, it is necessary to flexibly apply a variety of examination methods to confirm the diagnosis in clinical practice. Initial diagnosis can usually be made by imaging studies, such as CT scan and upper gastrointestinal radiography, followed by gastroscopy and treatment after adequate preparation. Kwon et al^[35]^ reported the 1st case of IED 1st diagnosed by transabdominal ultrasound. This suggests that careful evaluation of the esophagogastric junction or distal esophagus makes it possible to accurately diagnose lower or full IED by transabdominal USG. According to the patient’s condition, the comprehensive application of relevant examinations is very important for the diagnosis of suspected IED patients.

The majority of IED can be treated by conservative treatment, such as fasting water, enteral nutrition, acid-suppressive drugs, and antibiotics. However, some cases need surgical treatment because of severe complications, such as persistent dysphagia, mediastinal emphysema, and abscess formation. Endoscopic treatment is the most common method of invasive treatment. The purpose of eliminating the false lumen was achieved by cutting the dissection interval between the true and false lumen of the esophagus.^[14,28]^ Chen et al^[48]^ successfully closed the false lumen by making the false lumen mucosa adhere and heal by means of negative pressure suction. On the 1 hand, stent implantation can close the false lumen, promote mucosal healing and prevent the occurrence of serious complications.^[34,46]^ At the same time, it is also one of the optional treatments for some patients with severe esophageal stenosis. The extent of mucosal injury is related to the severity, treatment and prognosis of IED. Most patients with spontaneous IED develop partial dissection, whereas circumferential IED may be a more severe type of IED. Seong Hun Kim et al^[1]^ reported a case of IED with circumferential esophageal submucosal tear. The diagnosis and treatment of this case included conservative treatment and endoscopic mucosal septal incision, but the patient developed severe esophageal stenosis and was treated by endoscopic esophageal stent placement. A small number of cases require surgical treatment. These patients usually have severe dissection, such as circumferential dissection or esophageal mucosal prolapse, or have serious complications, such as esophageal perforation, mediastinal emphysema, and mediastinal abscess.

This case was an “accidental” finding of IED. During gastroscopy, the patient was once diagnosed with “esophageal fistula” due to the lack of understanding of the disease by endoscopists. Esophageal perforation and fistula were excluded after chest CT scan examination. According to the endoscopic findings and literature review, the final diagnosis was IED. The underlying disease of this patient was decompensated cirrhosis, combined with thrombocytopenia and abnormal coagulation function, which may be the potential cause of esophageal intramural dissection in this patient.

## 4. Conclusion

This case represents the 1st reported instance of asymptomatic IED discovered incidentally during a routine gastroscopy at our institution. The patient’s underlying decompensated liver cirrhosis, along with thrombocytopenia and abnormal coagulation function, likely contributed to the development of IED. This case emphasizes the importance of considering IED in differential diagnoses even in asymptomatic patients. Conservative management proved effective and may be considered a viable option in similar cases. Sharing such case reports enhances our understanding and management of IED, potentially improving outcomes for future patients.

## Author contributions

**Conceptualization:** Gong Cheng, Xiaoguo Wei.

**Data curation:** Li Wang, Jiateng You, Liangliang Cheng.

**Formal analysis:** Liangliang Cheng.

**Investigation:** Gong Cheng, Li Wang.

**Methodology:** Liangliang Cheng, Fan Yang, Fang He.

**Project administration:** Xiaoguo Wei.

**Resources:** Fan Yang.

**Software:** Fang He.

**Supervision:** Changjin Li.

**Validation:** Xiaolu Wang, Changjin Li.

**Writing – original draft:** Gong Cheng, Li Wang.

**Writing – review & editing:** Gong Cheng, Xiaoguo Wei.

## Supplementary Material


