# Supplementary material for: Asymptomatic intramural esophageal dissection: A case report and literature review
Source: Medicine (Baltimore). 2025 May 30;104(22):e42632. doi: 10.1097/MD.0000000000042632 (PMC12129498; doi:10.1097/MD.0000000000042632)
Supplement: Supplementary file 1 [file medi-104-e42632-s001.docx]

| Study | Year | Age | Sex | Location | Etiology and inducement | Therapy | Symptom | Tests to confirm the diagnosis |
| --- | --- | --- | --- | --- | --- | --- | --- | --- |
| Morritt^[1]^ | 1980 | 68 | F | thoracic | spontaneous | conservative treatment and intravenous fluids | chest pain, odynophagia, hematemesis | upper gastrointestinal radiography |
| Yeoh^[2]^ | 1985 | 70 | M | thoracic | spontaneous | conservative treatment | chest pain, odynophagia | upper gastrointestinal radiography |
| Takaoka^[3]^ | 1993 | 87 | F | thoracic, abdominal | nasobiliary catheter placement | conservative treatment, blood transfusion | hematemesis | gastroscopy |
| Phan^[4]^ | 1997 | 76 | F | NA | warfarin therapy | conservative treatment, nasogastric drainage, analgesic therapy, intravenous fluids, and intravenous antibiotics | chest pain, epigastralgia | upper gastrointestinal radiography |
| Bak^[5]^ | 1998 | 59 | M | thoracic, abdominal | spontaneous | endoscopic dissection, intravenous antibiotics, and total parenteral nutrition | chest pain, dysphagia, hematemesis, fever | CT |
| Hsu^[6]^ | 2001 | 41 | F | thoracic, abdominal | nasogastric tube insertion | conservative treatment, intravenous fluids | chest pain, dysphagia, odynophagia | CT |
| Cho^[7]^ | 2002 | 39 | M | thoracic, abdominal | drink alcohol | conservative treatment, intravenous antibiotics, and total parenteral nutrition | hematemesis, dysphagia, odynophagia | upper gastrointestinal radiography |
| Kim^[8]^ | 2005 | 46 | F | thoracic | prolonged prothrombin time and low platelet count due to liver cirrhosis | endoscopic dissection, balloon dilatation, and metal stent insertion | hematemesis, odynophagia | CT |
| Shelton^[9]^ | 2007 | 81 | M | thoracic | esophageal dilator | endoscopic dissection | dysphagia | gastroscopy |
| Liguori^[10]^ | 2008 | 32 | M | thoracic | eosinophilic esophagitis | a total esophagectomy with esophagogastroplasty and jejunostomy was performed | dysphagia, chest pain | gastroscopy |
| Hutchinson^[11]^ | 2008 | 77 | F | cervical, thoracic | nasogastric tube insertion | PPI | dysphagia | upper gastrointestinal radiography |
| Kim^[12]^ | 2009 | 62 | M | thoracic | spontaneous | endoscopic dissection | chest pain, dysphagia, odynophagia | gastroscopy |
| Krieg^[13]^ | 2009 | 45 | F | thoracic, abdominal | esophageal foreign body entrapment or upper endoscopy | esophagectomy with esophagogastroplasty | chest pain | upper gastrointestinal radiography |
| Benatta^[14]^ | 2010 | 32 | M | cervical, thoracic | paryngeal abscess | endoscopic dissection, endoscopic dilatation, intravenous antibiotics, jejunostomy, total enteral feeding | dysphagia | gastroscopy |
| Beumer^[15]^ | 2010 | 79 | F | whole | enoxaparin and aspirin therapy | conservative treatment, intravenous antibiotics, and PPI | hematemesis, dysphagia, back pain | CT |
|  |  | 72 | F | whole | warfarin and clopidogrel therapy | placement of feeding jejunostomy and gastrostomy tube, analgesic therapy, intravenous fluids, intravenous antibiotics, parenteral nutrition, and drainage of the chest | chest pain, dysphagia, hematemesis | CT |
|  |  | 78 | F | whole | aspirin and low-molecular-weight heparin therapy | PPI, intravenous antibiotics, intravenous fluids, drainage of the chest | hematemesis, dysphagia, retching | CT |
| Wang S.^[16]^ | 2011 | 56 | F | thoracic, abdominal | spontaneous | exploratory thoracotomy, partial esophagectomy, gastroesophageal anastomosis, intravenous antibiotics | dysphagia, odynophagia,fever | gastroscopy |
| Predina^[17]^ | 2012 | 19 | M | abdominal | eosinophilic esophagitis | intravenous fluids, blood transfusion | retching, vomit, hematemesis, melena | gastroscopy |
| El Hajj^[18]^ | 2012 | 31 | M | thoracic | transesophageal echocardiography | esophageal stent insertion | chest pain, hematemesis | CT |
| Cho^[19]^ | 2012 | 37 | F | cervical | the transorally inserted anvil and insertion of nasogastric tubes | conservative treatment, intravenous fluids, intravenous antibiotics | sore throat | gastroscopy |
| Singh^[20]^ | 2012 | 72 | M | thoracic | gastroscopy | intravenous fluids | chest pain, dysphagia, hematemesis | CT |
| Sgrò^[21]^ | 2012 | 15 | M | thoracic | eosinophilic esophagitis | endoscopic dissection | chest pain, dysphagia, hematemesis | CT |
| Cha^[22]^ | 2012 | 46 | M | thoracic | spontaneous | conservative treatment, intravenous antibiotics, and total parenteral nutrition | chest pain, hematemesis | gastroscopy |
| Monu^[23]^ | 2013 | 76 | M | thoracic | vomiting | conservative treatment | retching, vomit | CT |
| Khil^[24]^ | 2014 | 54 | M | cervical, thoracic | spontaneous | partial esophagectomy, gastroesophageal anastomosis | chest pain, dysphagia | CT |
| Romano-Munive^[25]^ | 2014 | 21 | F | whole | gastroscopy | conservative treatment, intravenous fluids, intravenous antibiotics, and total parenteral nutrition | NA | gastroscopy |
| Wang X.^[26]^ | 2014 | 52 | F | thoracic | resection of leiomyoma through gastroscopy | conservative treatment, gastrointestinal decompression, PPI, infection prevention and nutritional support | NA | CT |
| Kwon^[27]^ | 2015 | 37 | M | thoracic, abdominal | spontaneous | conservative treatment | chest pain, odynophagia, epigastralgia, fever | transabdominal ultrasonography |
| Fischer^[28]^ | 2015 | 43 | M | thoracic | gastroscopy | esophageal stents, jejunal feeding tubes, intravenous antibiotics, PPI | chest pain, dysphagia | CT |
| Ibáñez-Sanz^[29]^ | 2016 | 35 | M | thoracic, abdominal | eosinophilic esophagitis | conservative treatment, intravenous antibiotics, PPI, and parenteral nutrition | chest pain, retching, sialorrhea | CT |
| Ooi^[30]^ | 2016 | 18 | M | whole | spontaneous | endoscopic dissection | chest pain, dysphagia | CT |
| Zhu^[31]^ | 2016 | 37 | M | whole | spontaneous | esophageal stents, intravenous antibiotics, drainage of the chest, partial esophagectomy, gastroesophageal anastomosis, and nasal feeding | dyspnea, right-sided chest discomfort | CT |
| Wang^[32]^ | 2016 | 78 | M | NA | gastroscopy | conservative treatment | hematemesis, chest pain, dysphagia | gastroscopy |
|  |  | 54 | M | NA | gastroscopy | conservative treatment | hematemesis, chest pain, vomit | gastroscopy |
|  |  | 61 | F | NA | gastroscopy | conservative treatment | hematemesis, chest pain, vomit | gastroscopy |
| Özçınar^[33]^ | 2016 | 86 | F | thoracic | low-molecular-weight heparin therapy and vomit | conservative treatment, total parenteral nutrition | chest pain, hematemesis, back pain, odynophagia | gastroscopy |
| Wu^[34]^ | 2016 | 50 | M | cervical | vomiting | surgical resection | repeated vomiting presented with a giant smelly tube-like mass hanging from mouth | CT |
| Lee^[35]^ | 2017 | 77 | F | thoracic, abdominal | spontaneous | conservative treatment, nasal feeding | epigastralgia, poor intake of food, and progressive confusion for 1 week | CT |
| Yang^[36]^ | 2017 | 61 | F | Whole esophagus and gastric fundus | spontaneous | PPI, intravenous antibiotics, and total parenteral nutrition | chest pain, odynophagia, dysphagia, epigastralgia | NA |
| Wong^[37]^ | 2018 | 51 | M | cervical | vomiting | conservative treatment, PPI, intravenous antibiotics | vomit, fever, productive cough, odynophagia, dysphagia | CT |
| Castaninha^[38]^ | 2018 | 11 | M | NA | eosinophilic esophagitis | conservative treatment, enteral nutrition, PPI (PPI), predniso- lone, intravenous antibiotics | chest pain, dysphagia, odynophagia, sialorrhea | gastroscopy |
| Zhou^[39]^ | 2018 | 40 | M | thoracic, abdominal | ingesting coarse food | metal stent insertion | dysphagia, hematemesis | gastroscopy |
| Kim^[40]^ | 2018 | 2月 | M | cervical, thoracic | laryngeal mask airway | conservative treatment, PPI | fever, dysphagia and a symptom of aspiration with pneumonia | upper gastrointestinal radiography |
| Gao^[41]^ | 2018 | 56 | M | thoracic | spontaneous | conservative treatment, nasal feeding | choking after eating, hiccups, loss of appetite | gastroscopy |
| Chen^[42]^ | 2019 | 37 | M | thoracic, abdominal | spontaneous | Based on the gastroscopic two-tube system: negative pressure suction and enteral nutrition | chest pain, dysphagia, retching, loss of appetite | CT |
|  |  | 62 | M | cervical, thoracic | drink alcohol | Based on the gastroscopic two-tube system: negative pressure suction and enteral nutrition | odynophagia, sore throat | CT |
| Abdi^[43]^ | 2019 | 66 | F | thoracic | aspirin and clopidogrel therapy | conservative treatment | chest pain, dysphagia, odynophagia | CT |
| Ye^[44]^ | 2019 | 68 | F | abdominal | peroral endoscopic myotomy (POEM) | conservative treatment | NA | gastroscopy |
| Kumar^[45]^ | 2020 | 42 | M | thoracic | spontaneous | the incision and drainage of intramural oesophageal abscess | generalized fatigue, diffuse arthralgia, muscle cramps, and dark urine | CT |
| Anand^[46]^ | 2020 | 40 | M | whole | vomiting | trans hiatal esophagectomy (THE) and gastric pull through | vomit, chest pain, dysphagia, nasal regurgitation of food | CT |
| Hu^[47]^ | 2021 | 75 | M | thoracic, abdominal | spontaneous | endoscopic dissection | difficulty in eating, choking, and soreness in the chest and back | CT |
| Umehara^[48]^ | 2022 | 44 | F | cervical, thoracic | eosinophilic esophagitis | conservative treatment, intravenous antibiotics, PPI, and parenteral nutrition | dysphagia, odynophagia, neck pain | CT |
| Pajot^[49]^ | 2022 | 42 | F | cervical, thoracic | chest compressions and dual antiplatelet therapy (aspirin and clopidogrel therapy) | conservative treatment | chest pain, hematemesis | gastroscopy |

Supplementary Table 1. Characteristic of patients, location, etiology, treatment, and diagnostic examination methods in the cases of IED. PPI: Proton Pump Inhibitor; CT: Computed tomography; POEM: peroral endoscopic myotomy.

**Reference**

1. Morritt, G.N. and P.R. Walbaum, *Spontaneous dissection of the oesophagus.* Thorax, 1980. **35**(12): p. 898-900.

2. Yeoh, N.T., et al., *Intramural rupture and intramural haematoma of the oesophagus.* Br J Surg, 1985. **72**(12): p. 958-60.

3. Takaoka, M., et al., *Intramural rupture of the esophagus: a rare complication associated with nasobiliary catheter placement.* Intern Med, 1993. **32**(2): p. 185-8.

4. Phan, G.Q. and R.F. Heitmiller, *Intramural esophageal dissection.* The Annals of Thoracic Surgery, 1997. **63**(6): p. 1785-1786.

5. Bak, Y.T., et al., *Endoscopic treatment in a case with extensive spontaneous intramural dissection of the oesophagus.* Eur J Gastroenterol Hepatol, 1998. **10**(11): p. 969-72.

6. Hsu, C.C. and C.S. Changchien, *Endoscopic and radiological features of intramural esophageal dissection.* Endoscopy, 2001. **33**(4): p. 379-381.

7. Cho, C.-M., et al., *Endoscopic incision of a septum in a case of spontaneous intramural dissection of the esophagus.* Journal of Clinical Gastroenterology, 2002. **35**(5): p. 387-390.

8. Kim, S.H. and S.-O. Lee, *Circumferential intramural esophageal dissection successfully treated by endoscopic procedure and metal stent insertion.* Journal of Gastroenterology, 2005. **40**(11): p. 1065-1069.

9. Shelton, J.H., D.B. Mallat, and S.J. Spechler, *Esophageal obstruction due to extensive intramural esophageal dissection: diagnosis and treatment using an endoscopic 'rendezvous' technique.* Diseases of the Esophagus: Official Journal of the International Society for Diseases of the Esophagus, 2007. **20**(3): p. 274-277.

10. Liguori, G., et al., *Circumferential mucosal dissection and esophageal perforation in a patient with eosinophilic esophagitis.* World J Gastroenterol, 2008. **14**(5): p. 803-4.

11. Hutchinson, R., A.R. Ahmed, and D. Menzies, *A case of intramural oesophageal dissection secondary to nasogastric tube insertion.* Ann R Coll Surg Engl, 2008. **90**(7): p. W4-7.

12. Kim, E.S., et al., *Intramural esophageal dissection resolved by endoscopic treatment.* Endoscopy, 2009. **41 Suppl 2**: p. E313-314.

13. Krieg, A., et al., *Combined esophageal injury complicated by progression to a second perforation: a case report.* J Med Case Rep, 2009. **3**: p. 9213.

14. Benatta, M.A., et al., *Intramural esophageal dissection due to pharyngeal abscess treated by endoscopic esophageal transection: a case report.* Gastroenterologie Clinique Et Biologique, 2010. **34**(4-5): p. 329-331.

15. Beumer, J.D., P.G. Devitt, and S.K. Thompson, *Intramural oesophageal dissection.* ANZ J Surg, 2010. **80**(1-2): p. 91-5.

16. Fa-bing, W.S.-h.L., et al., *Diagnosis and Treatment for Intramural Esophageal Dissection: Report of One Case and Literature Review.* Chinese Journal of Clinical Thoracic and Cardiovascular Surgery, 2011. **18**(5): p. 417-421.

17. Predina, J.D., et al., *Intramural esophageal dissection in a young man with eosinophilic esophagitis.* Annals of Thoracic and Cardiovascular Surgery: Official Journal of the Association of Thoracic and Cardiovascular Surgeons of Asia, 2012. **18**(1): p. 31-35.

18. El Hajj, I.I., L.P. Luz, and N.F. Fayad, *Intramural esophageal dissection after trans-esophageal echocardiogram.* Digestive and Liver Disease: Official Journal of the Italian Society of Gastroenterology and the Italian Association for the Study of the Liver, 2012. **44**(10): p. 883.

19. Cho, H.H., et al., *Iatrogenic intramural esophageal dissection secondary to insertion of nasogastric tubes and the transorally inserted anvil during robot-assisted total gastrectomy.* Korean Journal of Anesthesiology, 2012. **63**(3): p. 284-285.

20. Singh, A. and M. Papper, *Extensive intramural esophageal dissection: an unusual endoscopic complication.* Gastrointestinal Endoscopy, 2012. **75**(1): p. 186-187; discussion 187.

21. Sgrò, A., et al., *An unusual complication of eosinophilic esophagitis in an adolescent: intramural esophageal dissection.* Endoscopy, 2012. **44 Suppl 2 UCTN**: p. E419-420.

22. Cha, I.H., et al., *[A case of conservatively resolved intramural esophageal dissection combined with pneumomediastinum].* The Korean Journal of Gastroenterology = Taehan Sohwagi Hakhoe Chi, 2012. **60**(4): p. 249-252.

23. Monu, N.C. and B.L. Murphy, *Intramural esophageal dissection associated with esophageal perforation.* Rhode Island Medical Journal (2013), 2013. **96**(7): p. 44-46.

24. Khil, E.K., H. Lee, and K. Her, *Spontaneous intramural full-length dissection of esophagus treated with surgical intervention: multidetector CT diagnosis with multiplanar reformations and virtual endoscopic display.* Korean Journal of Radiology, 2014. **15**(1): p. 173-177.

25. Romano-Munive, A.F., G. Grajales-Figueroa, and J.A. Rumoroso-García, *Intramural esophageal dissection caused by upper endoscopy.* QJM: monthly journal of the Association of Physicians, 2014. **107**(12): p. 1047-1048.

26. Hai-hong, W.X.-j.X. and C. Gong-wen, *A case report of esophageal perforation with intramural dissection caused by endoscopic treatment.* Chinese Journal of Clinical Rational Drug Use, 2014(30): p. 168-169.

27. Kwon, L.M., et al., *Intramural esophageal dissection diagnosed on transabdominal ultrasonography.* Japanese Journal of Radiology, 2015. **33**(12): p. 764-768.

28. Fischer, A., J. Höppner, and H.-J. Richter-Schrag, *First successful treatment of a circumferential intramural esophageal dissection with perforation in a patient with eosinophilic esophagitis using a partially covered self-expandable metal stent.* Journal of Laparoendoscopic & Advanced Surgical Techniques. Part A, 2015. **25**(2): p. 147-150.

29. Ibáñez-Sanz, G., L. Rodríguez-Alonso, and N.M. Romero, *Spontaneous intramural esophageal dissection: an unusual onset of eosinophilic esophagitis.* Revista Espanola De Enfermedades Digestivas: Organo Oficial De La Sociedad Espanola De Patologia Digestiva, 2016. **108**(3): p. 156-157.

30. Ooi, M. and I. Norton, *Spontaneous intramural esophageal dissection successfully treated by endoscopic needle-knife incision.* Gastrointestinal Endoscopy, 2016. **84**(1): p. 195-196.

31. Zhu, R.Y., et al., *Spontaneous circumferential intramural esophageal dissection complicated with esophageal perforation and esophageal-pleural fistula: a case report and literature review.* Diseases of the Esophagus: Official Journal of the International Society for Diseases of the Esophagus, 2016. **29**(7): p. 872-879.

32. Wang, Q., et al., *Intramural esophageal dissection: a rare complication of upper gastrointestinal endoscopy.* QJM: monthly journal of the Association of Physicians, 2016. **109**(1): p. 71-72.

33. Özçınar, B., et al., *Esophageal transection.* Ulusal Cerrahi Dergisi, 2016. **32**(4): p. 281-284.

34. Wu, X.-N., et al., *Complete Intramural Esophageal Dissection Caused by Violent Vomiting.* The Annals of Thoracic Surgery, 2016. **102**(2): p. e159.

35. Lee, M.-H., I.C. Wu, and J.-Y. Lee, *Spontaneous intramural esophageal dissection mimicking esophageal rupture.* The Kaohsiung Journal of Medical Sciences, 2017. **33**(2): p. 102-103.

36. Yang, L., R. Liao, and H. Zhang, *Radiologic and endoscopic findings of intramural esophageal dissection combined with gastric mucosal dissection.* VideoGIE: An Official Video Journal of the American Society for Gastrointestinal Endoscopy, 2017. **2**(11): p. 295-296.

37. Wong, Y.M., et al., *Temporal Evolution of Intramural Esophageal Dissection with 3D Reconstruction and Cinematic Virtual Fly-Through.* Journal of Radiology Case Reports, 2018. **12**(2): p. 11-17.

38. Castaninha, S., J. Lopes, and A.I. Lopes, *Intramural esophageal dissection. A rare occurrence in pediatric eosinophilic esophagitis.* Revista Espanola De Enfermedades Digestivas: Organo Oficial De La Sociedad Espanola De Patologia Digestiva, 2018. **110**(7): p. 468-469.

39. Zhou, B., et al., *Dysphagia and hematemesis caused by an intramural esophageal dissection.* Revista Espanola De Enfermedades Digestivas: Organo Oficial De La Sociedad Espanola De Patologia Digestiva, 2018. **110**(5): p. 327-328.

40. Kim, H.Y., et al., *Iatrogenic Intramural Dissection of the Esophagus after Insertion of a Laryngeal Mask Airway.* Acute Crit Care, 2018. **33**(4): p. 276-279.

41. Fei, G., C. Liang, and L. Leilei, *Intramural esophageal dissection: a case report and imaging diagnosis analysis.* Chinese Journal of Radiology, 2018. **52**(10): p. 797.

42. Chen, P.-H., S.-M. Yang, and P.-M. Huang, *Management of Patients With Circumferential Intramural Esophageal Dissection.* The Annals of Thoracic Surgery, 2019. **108**(1): p. e55-e56.

43. Abdi, S., et al., *Intramural Esophageal Dissection: A Rare Cause of Acute Chest Pain after Percutaneous Coronary Intervention.* The Journal of Tehran Heart Center, 2019. **14**(3): p. 138-140.

44. Ye, L., et al., *Intramural esophageal dissection during peroral endoscopic myotomy.* Endoscopy, 2019. **51**(7): p. E197-E198.

45. Kumar, S., M.K. Sakthivel, and T. Bosemani, *Intramural Esophageal Abscess Complicated with Pleural Fistula: A Case Report.* Cureus, 2020. **12**(2): p. e6846.

46. Anand, S., *Circumferential intramural esophageal dissection with large mucosal defect.* Clinical Journal of Gastroenterology, 2020. **13**(4): p. 1-3.

47. Hu, J.-W., et al., *Rare spontaneous extensive annular intramural esophageal dissection with endoscopic treatment: A case report.* World Journal of Clinical Cases, 2021. **9**(36): p. 11467-11474.

48. Umehara, M., et al., *Intramural esophageal dissection with eosinophilic esophagitis.* Clinical Journal of Gastroenterology, 2022. **15**(4): p. 681-687.

49. Pajot, G. and A.M. Lipowska, *Intramural Esophageal Dissection After Cardiopulmonary Resuscitation.* Clinical Gastroenterology and Hepatology: The Official Clinical Practice Journal of the American Gastroenterological Association, 2022. **20**(1): p. e4.
